# Primary Cutaneous Mucinous Carcinoma of the Eyelid

**Published:** 2017-09-27

**Authors:** Katherine Smith, Jake Laun, Wilton Triggs, Wyatt G. Payne

**Affiliations:** University of Central Florida College of Medicine, Orlando; and Division of Plastic Surgery, Department of Surgery, University of South Florida Morsani College of Medicine, Tampa

**Keywords:** eyelid, mucinous carcinoma, sweat gland, adenocarcinoma, malignant

## DESCRIPTION

**Figure 1:**
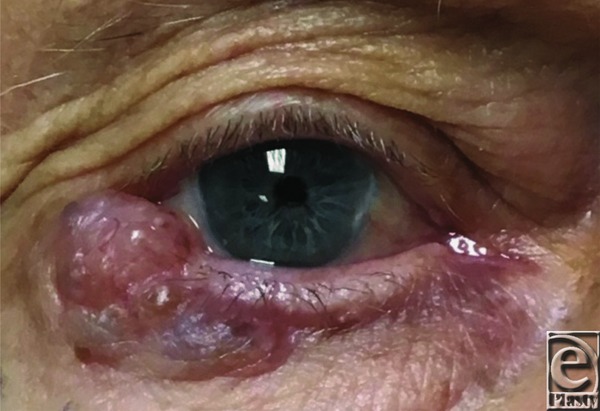
Gross image of eyelid lesion.

**Figure 2:**
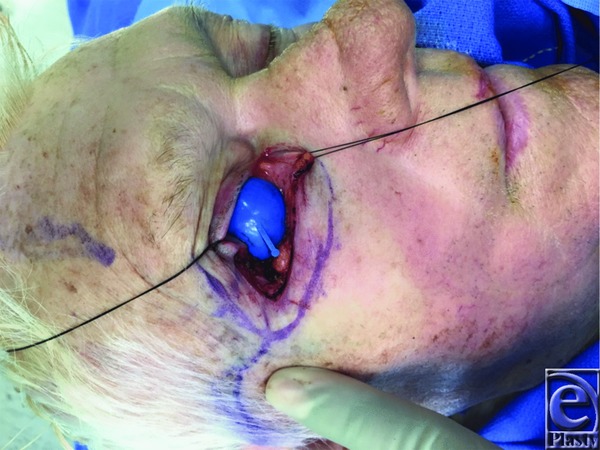
Intraoperative resection.

**Figure 3:**
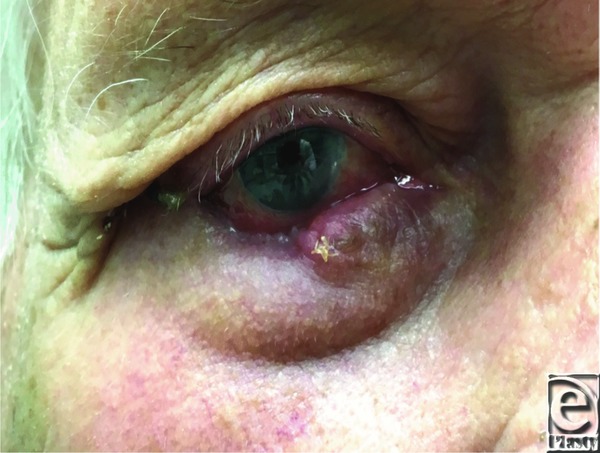
Early postoperative image.

An 89-year-old man presented with a right eyelid lesion of several months’ duration. The lesion measured 1×2 cm and involved one-third of the lower eyelid, including the lateral canthus (Fig 1). The tumor initially resembled a basal cell carcinoma; however, after excision, pathology revealed the lesion to be a primary mucinous carcinoma (Figs 2 and 3).

## QUESTIONS

1. What is mucinous carcinoma of the eyelid?
2. How is it diagnosed?
3. How is it treated?
4. What is the prognosis?

## DISCUSSION

Primary cutaneous mucinous carcinoma is a rare malignant neoplasm of the skin that originates from sweat glands. These lesions have been reported under several names, including adenocystic, colloid, gelatinous, and mucinous eccrine carcinoma, with the latter being a misnomer, as current evidence supports origination from apocrine differentiation.^1^ The majority of these tumors arise on the face, with 30% on the eyelid and 43% elsewhere on the head and neck. These tumors are characterized by an indolent course of local growth over months to years, usually in older male patients. The pattern of growth is often horizontal onto the tarsal plate compared with the vertical growth pattern of sebaceous tumors.^2^ Grossly, these tumors present as a painless papular or nodular lesion, occasionally with ulceration or crusting.^3^ They often resemble a cyst, basal cell carcinoma, chalazion, keratoacanthoma, or nevus.

**Figure 4:**
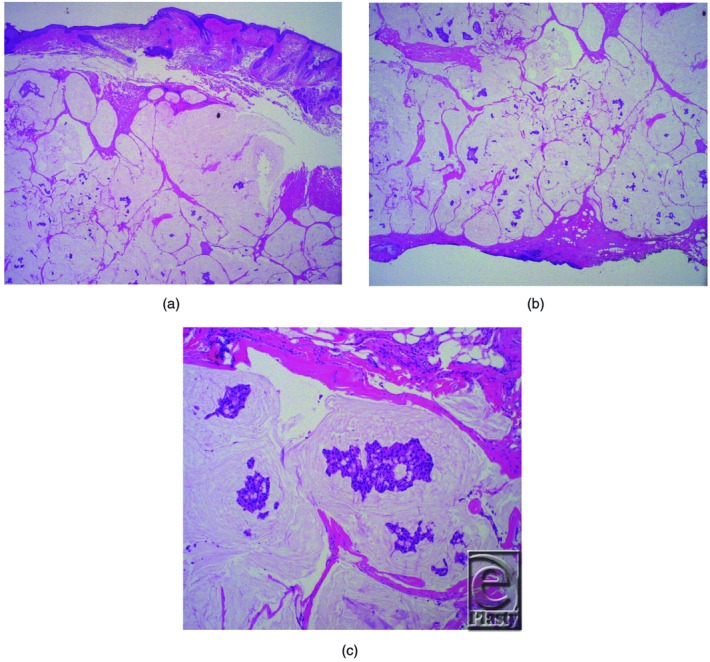
(a-c) Pathology specimens demonstrating pools of extracellular mucin with islands of tumor showing pleomorphic nucleoli with irregular borders.

Because these tumors often cannot be identified clinically due to their close resemblance to other carcinomas, primary mucinous carcinoma of the eyelid is almost always diagnosed histologically. They appear as nests of cuboidal cells suspended in pools of sialomucin. Cytologically, they display a low mitotic count and little nuclear atypia. These features are consistent with the low-grade nature of this lesion. Several classifications of primary mucinous carcinoma exist, most notably the endocrine mucin-producing carcinoma variant. The current literature is mixed regarding the significance of this variant, as it does not seem to have prognostic significance.^4^ A variety of immunohistochemical markers including CK7, CK20, and p63 are employed to distinguish these tumors from metastases of breast or gastrointestinal malignancies.^1^ These analyses were not performed in our case because the patient did not endorse any symptoms of an underlying malignancy, nor did he have a history of cancer. Figures 4*a*–4*c* depict pathology from our case.

Primary mucinous carcinoma of the eyelid is treated with surgical excision. Generous margins of 1.5 to 2.0 cm are preferred to reduce the likelihood of local recurrence.^5^ However, this is often impractical on the eyelid due to a limited quantity of nearby skin for reconstruction and the necessity to maintain aesthetics and function. Successful resection has been reported with narrow margins. The use of Mohs or frozen sections may allow for tighter control of margins, preserving neighboring tissue. If a large resection is unavoidable, reconstruction can be achieved with a Mustarde flap or other reconstructions, as appropriate.^6^^,^^7^ Our patient's older age and poor overall health prompted us to choose a simple local tissue rearrangement over a large reconstructive effort, with final pathology showing complete, successful resection with adequate functional and aesthetic outcomes.

Primary mucinous carcinomas are low-grade tumors but can be locally destructive, a concern considering their location and the complexity of reconstruction in this area. In addition, these lesions have a propensity for local recurrence, with recurrence rates as high as 40%. Burris et al^8^ described a case with local recurrences requiring reexcision over the course of 30 years. The ability to metastasize to lymph nodes and distant tissues has been reported, but these cases are exceedingly rare. Primary mucinous carcinoma is sufficiently rare to warrant consideration of investigation for underlying visceral malignancy, namely, of the breast and gastrointestinal tract.^9^

Primary cutaneous mucinous carcinoma of the eyelid is a rare neoplasm arising from sweat glands. These tumors often resemble common lesions, thus are often unsuspected until confirmed by tissue diagnosis. They are characterized by slow growth and local invasion with a high rate of recurrence. The preferred treatment is local excision with wide margins, but the location of these tumors often requires more narrow margins with reconstructive efforts depending on the extent and location of the tumor.
